# Phenolic Compounds Present *Schinus terebinthifolius* Raddi Influence the Lowering of Blood Pressure in Rats

**DOI:** 10.3390/molecules22101792

**Published:** 2017-10-23

**Authors:** Lorena de Lima Glória, Mariana Barreto de Souza Arantes, Silvia Menezes de Faria Pereira, Guilherme de Souza Vieira, Camilla Xavier Martins, Almir Ribeiro de Carvalho Junior, Fernanda Antunes, Raimundo Braz-Filho, Ivo José Curcino Vieira, Larissa Leandro da Cruz, Douglas Siqueira de Almeida Chaves, Silvério de Paiva Freitas, Daniela Barros de Oliveira

**Affiliations:** 1 Laboratório de Tecnologia de Alimentos, CCTA, Universidade Estadual do Norte Fluminense Darcy Ribeiro, Campos dos Goytacazes 28013-602, Brazil; lorena_limagloria@hotmail.com (L.d.L.G.); mariana.arant@yahoo.com.br (M.B.d.S.A.); silvia@uenf.br (S.M.d.F.P.); larissa.leandrocruz@gmail.com (L.L.d.C); 2 Laboratório de Clínica e Cirurgia Animal, CCTA, Universidade Estadual do Norte Fluminense Darcy Ribeiro, Campos dos Goytacazes 28013-602, Brazil; guilhermesv.medvet@gmail.com (G.d.S.V.); camilla.xm@gmail.com (C.X.M.); prfernandaantunes@yahoo.com.br (F.A.); 3 Laboratório de Ciências Químicas, CCT, Universidade Estadual do Norte Fluminense Darcy Ribeiro, Campos dos Goytacazes 28013-602, Brazil; almir@uenf.br (A.R.d.C.J.); braz@uenf.br (R.B.-F.); curcino@uenf.br (I.J.C.V.); 4 Laboratório de Química de Bioativos Naturais, Departamento de Ciências Farmacêuticas, Universidade Federal Rural do Rio de Janeiro, Seropédica 23897-000, Brazil; chavesdsa@yahoo.com.br; 5 Laboratório de Fitotecnia, Universidade Estadual do Norte Fluminense Darcy Ribeiro, Campos dos Goytacazes 28013-602, Brazil; silverio@uenf.br

**Keywords:** *Schinus terebinthifolius* Raddi, Anacardiaceae, naringenin, gallic acid, blood pressure, rotarod

## Abstract

This study identified two phenolic compounds in *Schinus terebinthifolius* Raddi fruits: naringenin (first report in this species) and gallic acid. Their structures were elucidated by nuclear magnetic resonance (NMR) data (^1^H-, ^13^C-NMR) and a high-performance liquid chromatography (HPLC) technique. A high content of phenolics (659.21 mg of gallic acid equivalents/g of sample—Folin-Ciocalteau method) and total flavonoids (140.69 mg of rutin equivalents/g of sample—aluminum chloride method) were quantified in *S. terebinthifolius*, as well as high antioxidant activity (77.47%—2,2-diphenyl-1-picrylhydrazyl, DPPH method). The antihypertensive activity related to its phenolic content was investigated. After intravenous infusion in Wistar rats, these phenolics significantly reduced (*p* < 0.05) the systolic, median, and diastolic arterial pressures of individuals. The rotarod test was performed to determine the mechanism of action of the sample vasorelaxant effect. It was found that its action exceeded that of the positive control used (diazepam). This confirmed the vasodilatory activity exerted by *S. terebinthifolius* fruits is related to the phenolic compounds present in the plant, which are potent antioxidants and inhibit oxidative stress, mainly in the central nervous system.

## 1. Introduction

The World Health Organization (WHO) reported that about three-quarters of the world’s population make use of medicinal plants to improve health [1]. Medicinal plants can be considered one of the oldest forms of health care [2,3].

*Schinus terebinthifolius* Raddi, Anacardiaceae, is a plant native to South America popularly known as Aroeira or Pink Pepper [4]. This species is one of 71 medicinal plants reported in the National Relation of Medicinal Plants (RENISUS), which are of interest to the Brazilian Unified Health System (SUS) [5]. This list was created to promote and acknowledge the popular use of medicinal plants with potential effect on primary health care [5].

The discovery of biological activities attributed to phenolic compounds has been widely reported in several studies and has led the scientific community to carry out epidemiological studies on the likely associations between phenolic compounds and reduced risk for various diseases [6,7]. Natural compounds are responsible for the protective effect of cells against oxidative damage caused by Reactive Oxygen Species (ROS) [8]. Excess ROS in the body can cause a number of complications [7,8]. Antioxidants, which are responsible for neutralizing the effects of ROS and the scavenging of free radicals, may favor cardiovascular parameters in individuals [9,10].

Considering the importance of phenolic compounds and aiming to identify new natural sources with high biological potential, the total phenolics and flavonoids were quantified and the antioxidant activity and cardiovascular activity of the phenolic compounds in *Schinus terebinthifolius* Raddi fruits was determined. The present work is the first report on flavonoid naringenin and the activities attributed to it in this species.

## 2. Results and Discussion

### 2.1. Identification and Structure Elucidation of the Major Compounds

**Figure 1:**
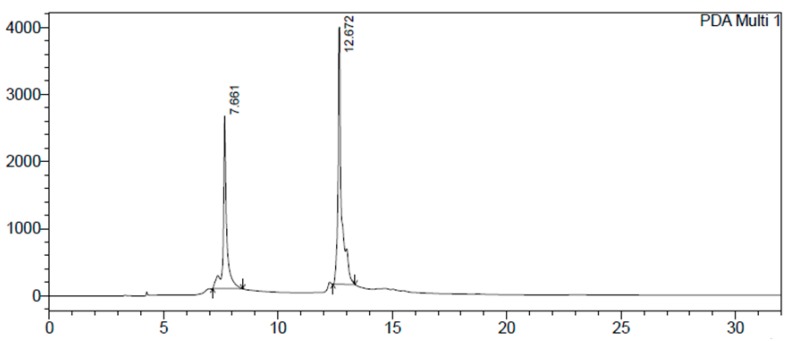
Chromatogram of the High Performance Liquid Chromatography with a Diode-Array Detector (HPLC-DAD) analysis of the investigated G5 fraction from *Schinus terebinthifolius* Raddi fruit extract (4.0 mg/mL) at wavelength of 254 nm. Analytical conditions: stationary phase: RP-18 reverse-phase column (5 μm, 250 mm, 4.5 mm, Macherey-Nagel); temperature: 32 °C; mobile phase: A = purified water adjusted to pH 3.2 with phosphoric acid, B = acetonitrile; flow rate: 1.0 mL/min; detection: 254 nm; injection volume: 20 μL; solvent composition during analysis: 0′: 100% A; 5′: 70% A; 10′: 50% A; 15′: 30% A; 20′: 20% A; 25′: 10% A; 30′: 0% A; 32′: stop.

The methanolic extract of *Schinus terebinthifolius* was partitioned into aqueous and organic fractions. The organic fraction was subjected twice to Sephadex LH-20 column chromatography to produce the G5 fraction, whose chemical profile was analyzed by High Performance Liquid Chromatography with a Diode-Array Detector (HPLC-DAD) (Figure 1).

The chromatogram shows two major peaks at retention times 7.7 and 12.7 min. Kanaze et al. (2004) validated a methodology for HPLC assay and identified naringenin at retention time 13.1 min, while Song et al. (2012) developed a methodology to determine phenols in HPLC and found gallic acid at the retention time 7.71 min [11,12]. 

Although Kanaze et al. (2004) and Song et al. (2012) have identified compounds with retention time close to those demonstrated in the present study, the authors did not use the same method of analysis developed by us, so without NMR analysis, we cannot be sure about identity of the analytes [11,12].

Although Kanaze et al. (2004) and Song et al. (2012) identified compounds with retention time close to those demonstrated in the present study, the authors did not use the same method of analysis developed by us, so without NMR analysis, we cannot be sure about identity of the analytes [11,12].

**Figure 2:**
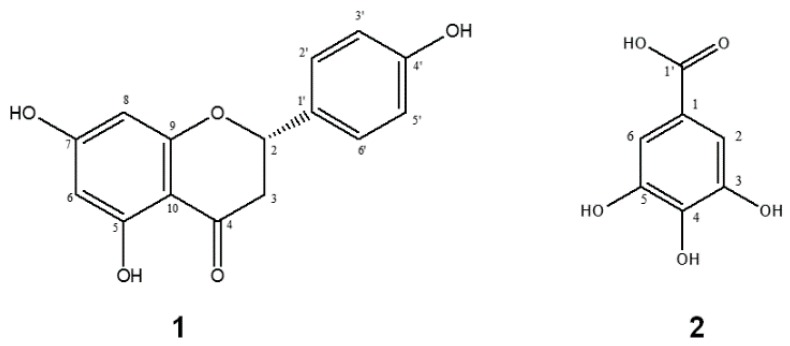
Chemical structures of compounds **1** and **2**.

The prevailing compounds present in the G5 fraction of *S. terebinthifolius* were identified, namely, a flavonoid and a phenolic acid: naringenin (**1**) and gallic acid (**2**) (Figure 2). The phenolic compounds were identified by NMR analysis (figures shown in Supplementary Material) sand comparing their ^1^H-, ^13^C-NMR spectral data with values in the literature [13,14]. Naringenin was reported for the first time in the fruits of this plant.

Compound **1** was obtained as yellow powder. ^1^H (DMSO-*d*_6_, 500 MHz): δ_H_ 7.22 (2H, d, H-2′ and H-6′), 6.70 (2H, d, H-3′ and H-5′), 5.89 (1H, s, H-6), 6.05 (1H, s, H-8), 5.56 (1H, dd *J* = 6.1 and 2.1 Hz, H-2), 3.16 (1H, m, H-3_a_), 2.80 (1H, br d *J* = 16.0 Hz, H-3_b_). ^13^C (DMSO-*d*_6_, 125 MHz): δ_C_ 196.9 (C-4), 167.1 (C-7), 164.0 (C-9), 163.6 (C-5), 157.9 (C-4′), 129.0 (C-1′), 128.2 (C-2′ and C-6′), 115.4 (C-3′ and C-5′), 102.2 (C-10), 96.2 (C-6), 95.4 (C-8), 79.1 (C-2), 42.8 (C-3). The data are in accordance with those published by Du et al. (2004) [13].

Compound **2** was obtained as yellow powder. ^1^H (DMSO-*d*_6_, 500 MHz): δ_H_ 6.92 (2H, s, H-2 and H-6). ^13^C (DMSO-*d*_6_, 125 MHz): δ_C_ 165.9 (C-1′), 145.9 (C-3 and C-5), 139.2 (C-4), 119.3 (C-1), 109.2 (C-2 and C-6). The data are in accordance with those published by Santana et al. (2012) [14]. 

Other researchers have identified gallic acid in leaves and stem bark of *S. terebinthifolius* [14,15], but there are no reports on the presence of naringenin flavanone in this species. The presence of I30′,II8-binaringenin in *S. terebinthifolius* was confirmed and allocated to the internal part of the fruit [16]. Some studies have identified only tetrahydrorobustaflavone [8] and tetrahydroamentoflavone [17] compounds in *S. terebinthifolius*, which are binaringenine linked at positions 6 and 5′, respectively, and I30,II8-binaringenin [18] and I3,II3-binaringenin [19] in fruits of *Schinus molle* have been reported. The present work is the first report on flavonoid naringenin and the activities attributed to it in this species.

### 2.2. Total Phenolic Compounds, Total Flavonoid Content and Antioxidant Activity

Natural compounds are responsible for the protective effect of cells against oxidative damage caused by Reactive Oxygen Species (ROS) [20]. Excess ROS in the body can cause a number of complications such as lipid peroxidation in cell membranes, aggression to proteins and enzymes and DNA damage, which can trigger pathological processes including cell aging, the onset of cancer, cardiovascular diseases, and other chronic diseases [6,7,20]. Antioxidants, which are responsible for neutralizing the effects of ROS, can be mainly found in compounds derived from secondary metabolites from plant sources, such as phenolic compounds [10,21]. Hence the need to determine the antioxidant activity and content of total flavonoids and phenolics in plants, since this is an important step for the identification of possible sources of bioactive molecules. 

Table 1 shows the content of total phenolic compounds, flavonoids and the antioxidant activity of the G5 fraction of *S. terebinthifolius* fruits using the Folin-Ciocalteau, aluminum chloride colorimetric assay and DPPH free radical methods, respectively. The results showed a significant phenolic content (over 650 mg Gallic Acid Equivalent (GAE)/g), a large amount of flavonoids (140.69 mg Rutin Equivalent (RE)/g), and high antioxidant activity (above 70%) for *Schinus terebinthifolius* fruits.

The content of total phenolics found in the Aroeira fruits used in the present study was significantly higher than that reported in the literature for the same species for fruit extract: 110 mg GAE/g [21], leaf extract: 384.64 mg GAE/g [22], extract from the stem: 309.03 mg GAE/g [21], and extract from the bark: 207 mg GAE/g [21]. A study proved that the maceration method was almost 20 times more effective than the soxhlet method, and the solvent used (methanol) was also the most effective to determine the content of total phenolics in Aroeira fruits [21].

Tabaldi et al. (2016) found a value lower than that of our work for total flavonoids in the methanolic extract from Aroeira leaves: 111.81 mg of quercetin equivalent (QE)/g extract [23]. Fedel-Miyasato et al. (2014) studied the same type of extract and reported a much higher value: 460.20 mg QE/g [24]. This value is higher than that found by Uliana et al. (2016) for ethanolic extract from leaves of *S. terebinthifolius*: 69.67 mg QE/g of macerated extract and 243.09 mg QE/g of extract obtained with the use of assisted ultrasound technique [20].

Generally, several assays have been frequently used to estimate antioxidant capacity in fruits and their products [25]. However, 2,2-diphenyl-1-picrylhydrazyl (DPPH) method measures the ability to scavenge free radicals. The test is simple, relatively rapid, reproducible, and does not require specialized equipment, and thus can be used for assessing antioxidant activity in foods and plant extracts [26].

Ethanolic extract from different parts of Aroeira obtained by maceration showed the following approximate antioxidant activities: fruits—60%, stem—90%, peels—70%, and leaves—30% [21]. The methanolic extract from Aroeira fruits showed antioxidant activity of 95.6% by the DPPH method in the work of Bernardes et al. (2014) [27]. El-Massry et al. (2009) evaluated the antioxidant activity of ethanolic extract, dichloromethane extract and essential oil from Aroeira leaves and observed that the ethanolic extract presented the highest antioxidant activity, with free radical sequestration above 80% [25].

Differences in the obtained values may be due to many factors. For example, climate conditions and fruit variety as well as processing methods (extraction, filtration, isolation), and storage conditions (air, temperature) can cause changes in the composition of phenols [8,28]. Furthermore, the antioxidant capacity of biomolecules is significantly influenced by the structure. The activity may be attributed to the enhanced stabilization of the radical state during electron transfer [10,20]. The present work is the first report on flavonoid naringenin and the activities attributed to it in this species.

### 2.3. In Vivo Blood Pressure Assessment 

**Figure 3:**
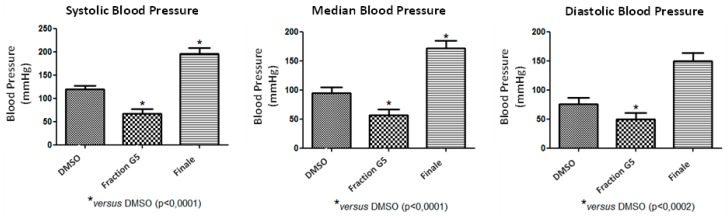
Effects of the G5 fraction (30 mg/kg) from *Schinus terebinthifolius* Raddi fruit extract on the blood pressure of Wistar rats. Systolic blood pressure (SBP), median blood pressure (MBP), diastolic blood pressure (DBP). The values are expressed as mean ± SEM from eight experiments. One-way ANOVA followed by Newman-Keuls test (* *p* < 0.05 compared to control).

Phenolic compounds are the main compounds related to antioxidant activity, and the scavenging of free radicals caused by this activity may favor cardiovascular parameters in individuals. An intravenous infusion of the G5 fraction was administered in rats to investigate if this sample may cause changes in blood pressure. After the administration of the infusion of G5, all parameters analyzed were significantly reduced compared to the final pressure and the control (DMSO) in Wistar rats at 5% probability (Figure 3). Comparison between pressure after G5 infusion and final pressure showed that the systolic pressure decreased by 128.3 mmHg, the median pressure decreased by 115.4 mmHg and the diastolic pressure decreased by 100.3 mmHg (*p* < 0.05). Comparison between G5 pressure and the DMSO control showed that the systolic pressure was reduced by 51.72 mmHg, the median pressure decreased by 37.83 mmHg and the diastolic pressure decreased by 26.35 mmHg (*p* < 0.05).

In this paper, it has been shown that G5 is a compound that induces blood pressure decrease in rats evaluated in vivo by intravenous administration. Hypertension is an important public health problem and is one of the most common cardiovascular diseases [29]. Phenolic compounds, which perform antioxidant activity, have already been associated with coronary vasodilator activity [9,29]. This stresses the relevance of developing new specific antihypertensive drugs with potential for clinical use.

The possible mechanisms by which phenolic compounds perform cardiovascular activity have led to studies on free radical sequestration, inhibition of lipid peroxidation, and enzymatic activities [9,30]. Phenols also inhibit platelet aggregation and the oxidation of LDL cholesterol and promote vasodilation, thus ensuring the integrity of blood vessels [9,31].

There are no reports in the literature on cardiovascular activity for any species of *Schinus* spp. However, for other species of the Anacardiaceae family, the following is described: aqueous extract of bark stem of *Anacardium occidentale* was administered intravenously in rabbits and significantly reduced arterial pressure and the contractile activity of the isolated heart of rat, thus presenting in vivo hypotensive activities and in vitro cardio-inhibitory activity [32]. Aqueous extract from the *Harpephyllum caffrum* stem significantly reduced systemic arterial pressures and heart rates in hypertensive rats after intravenous administration of the extract. The hypotensive effects are believed to be related to the presence of polyphenolic compounds and flavonoids in the plant [33]. Extracts from leaves of *Sclerocarya birrea* showed a significant antagonistic effect on calcium release due to the vasorelaxing effect observed in arterial vessels of rats, since calcium ions are responsible for the contraction of the smooth muscle [34].

**Figure 4:**
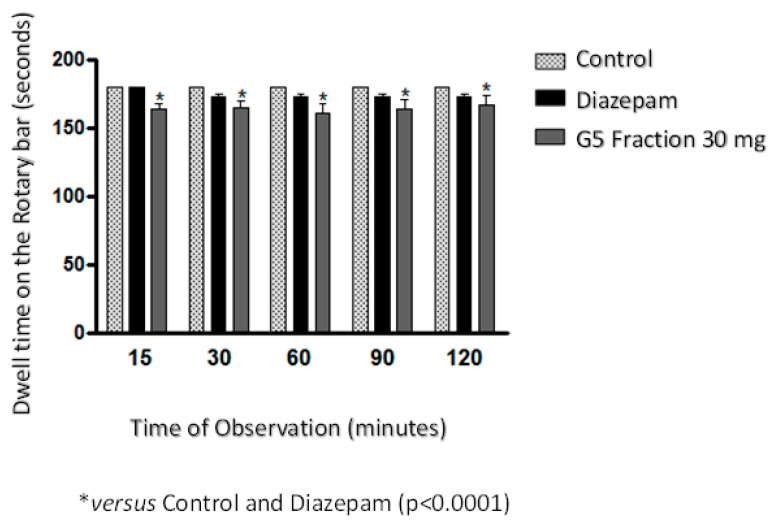
Effects of the G5 fraction (30 mg/kg) *Schinus terebinthifolius* Raddi fruit extract on Swiss mice (*Mus musculus*) in the Rotarod test. The values are expressed as means ± SEM of ten experiments. One-way ANOVA followed by Newman-Keuls test (* *p* < 0.05 compared to the control).

In the present study, it was also observed that the administration of the G5 fraction significantly affected (*p* < 0.0001) the motor incoordination of the mice (performance in rotarod) (Figure 4). As the time of permanence of G5 in the rotating bar was shorter than that of animals using diazepam, which is the positive control with activity on the central muscle relaxation, it is concluded that this sample has a more direct action on the central nervous system, with an effect on the peripheral musculature. If the time of permanence in the bar had been equal to that obtained with diazepam, the action would have been only on the peripheral plate.

Treatment with G5 produced hypolocomotion, increased immobility and muscular incoordination, with activity mainly observed on the central nervous system of the mice. These findings contradict previous studies reporting that the extract from the *S. terebinthifolius* stem bark alleviated the effects of rat motor incoordination caused by the administration of rotenone in the rotarod test [15], and that mangiferin isolated from the bark of *Mangifera indica* L. (Anacardiaceae) did not produce any significant effect on motor coordination in rotarod in rats indicating that the observed antinociception was not related to sedation or motor anomaly, but to the peripheral antinociceptive activity [35].

According to Seaman (2000), the locomotor impairment produced by G5 may be related to reduced energy levels and consequent changes in neural processing [36]. The brain is highly susceptible to free radical damage due to its high rate of oxygen utilization and the relatively low presence of antioxidant enzymes and free radical scavengers. There is growing interest in the establishment of therapeutic and dietary strategies to combat oxidative stress induced by damage mainly to the central nervous system [15]. Oxidative stress is a result of an imbalance between free radicals and may be ameliorated by the endogenous action of antioxidant defense systems [21]. Thus, the discovery of new natural sources rich in phenolic compounds and presenting antioxidant potential can prove the efficacy of the plants used in popular medicine, whose biological activities are scientifically proven. The present work is the first report on flavonoid naringenin and the activities attributed to it in this species.

## 3. Materials and Methods

### 3.1. General Experimental Procedures

^1^H- (500 MHz) and ^13^C- (125 MHz) NMR data were obtained on a Bruker Advance II 9.4 T instrument (Centro de Ciências e Tecnologia, UENF) using DMSO-*d*_6_ as solvent. Chromatographic purifications were carried out by using Sephadex LH-20 (25–100 μm, Sigma-Aldrich, St. Louis, MO, USA). High Performance Liquid Chromatography analyses were performed using a Shimadzu Prominence HPLC system with two LC10AT pumps, a scanning ultraviolet SPD-M10A photodiode array detector and a Rheodyne 7725i injector. The reverse-phase column used was an RP-18 (5 μm, 250 mm, 4.5 mm i.d., Macherey-Nagel, Bethlehem, PA, USA). Temperature: 32 °C. Mobile phase: A = purified water adjusted to pH 3.2 with phosphoric acid, B = acetonitrile was used as eluent. Flow elution rate was 1.0 mL/min; 20 μL of the samples (4 mg/mL) were injected, detection: 254 nm. Solvent composition during analysis: 0′: 100% A; 5′: 70% A; 10′: 50% A; 15′: 30% A; 20′: 20% A; 25′: 10% A; 30′: 0% A; 32′: stop. The UV/VIS spectrophotometer (Epoch—BioTek—versão: Gen 5 1:10, Winooski, VT, USA) was used. The hemodynamic parameters were measured using the Bioamp equipment (Adinstrumentes, Australia), and Graph Labsoftware (version 7.0; AD Instruments). The automated Rota Rod instrument (EFF 411, Insight^®^) was used.

### 3.2. Chemicals

Deuterated dimethyl sulfoxide (DMSO-*d*_6_ purity > 99%), Folin-Ciocalteu reagent, Butylated hydroxytoluene (BHT 99% purity), 2,2-Diphenyl-2-picryl hydrazyl (DPPH purity ≥ 99%), gallic acid and rutin, quercetin (purity of standards ≥ 99%) were all purchased from Sigma-Aldrich. Merck acetonitrile (HPLC purity ≥ 99.9%) was obtained from Merck. The anhydrous sodium carbonate (Na_2_CO_3_) was obtained from Synth. Aluminum chloride hexahydrate (AlCl_3_.6H_2_O) was purchased from VETEC. Isoflurane 100% and diazepam (5mg/mL) were obtained from Cristália, heparin 5000 IU/mL from Blaú—Cristal Pharma laboratory, sodium chloride (NaCl 0.9%) was purchased from Sanobiol. The other chemical reagents were purchased from VETEC. 

### 3.3. Animals 

The tests were performed on male and female Wistar rats (*Rattus norvegicus*) weighing between 250 and 300 g and male Swiss mice (*Mus musculus*) weighing between 25 and 30 g from the Animal Experimentation Unit of the Universidade Estadual do Norte Fluminense (UEA—UENF), which were kept in an environment with controlled temperature, 19 °C, humidity of 50 to 60%, and light/dark cycle of 12 h. Water and food were offered ad libitum. The present study was approved by the UENF Ethics Committee for Animal Use (CEUA), registered under protocol number 353. Each animal was used in only one experiment. 

### 3.4. Plant Material 

The fruits of *Schinus terebinthifolius* Raddi were collected in Campos dos Goytacazes, Rio de Janeiro State, Brazil (Latitude 21°44′ S and 41°18′ W; Altitude 12 m above sea level) in April 2014. A voucher specimen was identified and deposited at the UENF herbarium under the code H5073. 

### 3.5. Extraction and Isolation 

The fruits of *S. terebinthifolius* (250 g) were cleaned, washed, and subjected to methanol extraction (10% *w*/*v*) by static maceration for 30 days. Every seven days, the solvent was filtered through a paper filter (QUANTY number 41), placed to evaporate in water bath at 35 °C and protected from light [37] so as to obtain the crude extract (71.7 g). Then, the crude extract was partitioned with ethyl acetate resulting in two fractions: aqueous and organic. The organic fraction was rotary evaporated at 40 °C. The fractionation of the organic fraction (38.7 g) occurred in open Sephadex LH-20 Chromatographic Column using methanol as the eluent, which resulted in three fractions: G1 (7.6 g), G2 (19.4 g), and G3 (8.1 g). The G3 fraction was injected again into the Sephadex LH-20 column with methanol, and two more fractions were obtained: G4 (3.2 g) and G5 (4.4 g). The fraction G5 was the one used in the present work due to the profile shown in Thin Layer Chromatography (TLC) similar to flavonoids and phenolic compounds.

### 3.6. Total Phenolic Compounds 

The total phenolic compounds were determined by the Folin-Ciocalteau method previously described by Singleton et al. (1999) with modifications, using gallic acid calibration curve concentrations from 0 to 500 μg/mL [38]. An aliquot of 0.1 mL of sample diluted in methanol (1.0 mg/mL) was added in 1.0 mL of methanol and 0.1 mL of the Folin-Ciocalteau reagent. The mixture was homogenized and received the addition of 1.0 mL of Na_2_CO_3_ (7%) after 5 min. The reaction occurred for 90 min, in the dark, at room temperature. The absorbance was read at 760 nm and the results were expressed in milligrams of gallic acid equivalents (mg GAE/g) per gram of sample. 

### 3.7. Total Flavonoid Content 

Total flavonoids were established by a colorimetric assay described by Woisky and Salatino (1998) with modifications [39]. An aliquot of 7.5 mL of sample (0.1 mg/mL in methanol) was added to 0.5 mL of AlCl_3_ (5% *w*/*v*) and then to 25 mL of methanol. Absorbance reading was performed at 425 nm after 30 min at rest. The flavonoid content was calculated using rutin calibration curve concentrations from 0 to 100 mg/mL. The results were expressed in milligrams of rutin equivalents per gram of sample (RE/g). 

### 3.8. Antioxidant Activity 

The antioxidant activity was determined by the stable free radical DPPH method (2,2-diphenyl-1-picryl-hydrazyl) [26]. The samples were prepared in methanol at 2, 0.2 and 0.02 mg/mL. Samples (0.5 mL) were added to 0.5 mL methanolic solution of DPPH (4%) to final concentrations of 1, 0.1 and 0.01 mg/mL. The reaction was incubated in darkness, for 60 min at 25 °C. The absorbance values were measured at 515 nm. The radical scavenging activity (% inhibition) was expressed as percentage of scavenged DPPH and calculated according to the following equation: % of Inhibition = [(ADPPH − Asample)/ADPPH] × 100, where ADPPH is the absorbance of DPPH solution (negative control) and Asample is the absorbance of the sample in the presence of DPPH. BHT and quercetin were used as positive controls. 

### 3.9. In Vivo Blood Pressure Assessment 

Wistar rats were anesthetized by inhalation with isoflurane and contained for catheter insertion in the left carotid artery, through which the following parameters analyzed were measured: systolic, median, and diastolic blood pressure. The cannula was heparinized with a sodium heparin solution and 0.9% sodium chloride in order to avoid blood clotting. Another catheter was inserted into the jugular vein for intravenous infusion of the G5 fraction at a dose of 30 mg/kg diluted in DMSO with a volume of 0.1 mL per animal. Prior to the tests, DMSO alone was infused at the same dose to serve as a control so as to eliminate the hypotensive effects of DMSO on the results. 

### 3.10. Rotarod Test 

The Swiss mice (*Mus muscullus*) were previously tested on the rotating bar. Those that fell two or more times in three-minute period were discarded. After the selection of the animals, the G5 fraction and the diazepam positive control were administered intraperitoneally, 0.1 mL volume and 300 mg/mL concentration in DMSO.

Each individual was placed with all four legs on a rotating bar of 8 cm diameter, 20 cm from the bottom of the equipment, already in motion (20 rpm). The time they could balance before falling was measured. The mice were observed at the times of 15, 30, 60, 90, and 120 min after sample administration, and remained on the rotating bar for three minutes. At the fall, the chronometer used to verify the time of equilibrium would stop automatically, the animals returned to their respective bars, and the chronometer would be reactivated, so that the total falls would be counted after the three minutes, while a general timer measured the total time of the test (120 min). 

### 3.11. Statistical Analysis 

All experiments were performed in triplicates, and the results were expressed as mean ± standard deviation (SD). The results obtained were tabulated by the LabChart 7 program, and statistically analyzed through the GraphPad Prisma 5. The analysis of variance (ANOVA) was defined, followed by the Newman-Keuls and Bonferroni mean test, with reliability index of 95%. 

## 4. Conclusions

This study provides evidence for the pharmacological antihypertensive and antioxidant potential of *Schinus terebinthifolius* Raddi fruit extract. Phytochemical investigations identified two major compounds of the G5 fraction from methanolic extract: naringenin (first report in this species) and gallic acid, which were analyzed for the first time by inducing blood pressure decrease evaluated in vivo. Phenolic compounds present in *S. terebinthifolius* were identified as a promising natural source to combat cardiovascular and related diseases.

## Figures and Tables

**Table 1:** Total phenolic compounds, total flavonoid content, and antioxidant activity of G5 fraction from *Schinus terebinthifolius* Raddi fruit extract.

	Total Phenolic Compounds (mg GAE */g)	Total Flavonoid Content (mg RE **/g)	Antioxidant Activity (%)		
			**0.01 mg/mL**	**0.01 mg/mL**	**0.01 mg/mL**
Fraction G5	659.21 ± 6.05	140.69 ± 9.44	77.5 ± 2.57	75.0 ± 6.78	69.2 ± 6.47
Quercetin	1214.71 ± 9.51	1009.27 ± 7.85	77.2 ± 0.73	74.1 ± 3.82	71.0 ± 3.63
BHT ***			76.4 ± 1.98	75.0 ± 4.94	66.3 ± 4.20

Values are means of three determinations standard deviation. * GAE = Gallic Acid Equivalent, ** Rutin Equivalent, *** BHT = Butylated hydroxytoluene.

## Supplementary Materials

^1^H-, ^13^C- and ^13^C-DEPTQ NMR spectra, COSY, HSQC, and HMBC spectra correlations, and structures of compound **1** and **2** assembled with the aid of COSY correlations are available as Supplementary Materials available online.
