# Supplementary material for: Phenolic Compounds Present Schinus terebinthifolius Raddi Influence the Lowering of Blood Pressure in Rats
Source: Molecules. 2017 Oct 23;22(10):1792. doi: 10.3390/molecules22101792 (PMC6151430; doi:10.3390/molecules22101792)
Supplement: Supplementary file 1 [file molecules-22-01792-s001.pdf]

molecules

Supplementary Materials

Phenolic compounds in *Schinus terebinthifolius* Raddi influence the lowering of blood pressure in rats

Lorena de Lima Glória [1], Mariana Barreto de Souza Arantes [1], Silvia Menezes de Faria Pereira [1], Guilherme de Souza Vieira [2], Camilla Xavier Martins [2], Almir Ribeiro de Carvalho Junior [3], Fernanda Antunes [2], Raimundo Braz-Filho [3], Ivo José Curcino Vieira [3], Larissa Leandro da Cruz [1], Douglas Siqueira de Almeida Chaves [4], Silvério de Paiva Freitas [5] and Daniela Barros de Oliveira [1,*]

[1] Laboratório de Tecnologia de Alimentos, CCTA, Universidade Estadual do Norte Fluminense Darcy Ribeiro, Campos dos Goytacazes, RJ 28013-602, Brazil; lorena_limagloria@hotmail.com (L.d.L.G.); mariana.arant@yahoo.com.br (M.B.d.S.A.); silvia@uenf.br (S.M.d.F.P.); larissa.leandrocruz@gmail.com (L.L.d.C.); dbarrosoliveira@uenf.br (D.B.d.O.)
[2] Laboratório de Clínica e Cirurgia Animal, CCTA, Universidade Estadual do Norte Fluminense Darcy Ribeiro, Campos dos Goytacazes, RJ 28013-602, Brazil; guilhermesv.medvet@gmail.com (G.d.S.V.); camilla.xm@gmail.com (C.X.M.); prfernandaantunes@yahoo.com.br (F.A.)
[3] Laboratório de Ciências Químicas, CCT, Universidade Estadual do Norte Fluminense Darcy Ribeiro, Campos dos Goytacazes, RJ 28013-602, Brazil; almir@uenf.br (A.R.d.C.J.); braz@uenf.br (R.B.-F.); curcino@uenf.br (I.J.C.V.)
[4] Laboratório de Química de Bioativos Naturais, Departamento de Ciências Farmacêuticas, Universidade Federal Rural do Rio de Janeiro, RJ, Brazil chavesdsa@yahoo.com.br (D.S.d.A.C.)
[5] Laboratório de Fitotecnia, Universidade Estadual do Norte Fluminense Darcy Ribeiro, Campos dos Goytacazes, RJ 28013-602, Brazil; silverio@uenf.br (S.d.P.F.)
* Correspondence: dbarrosoliveira@uenf.br; Tel.: +55-22-27486517

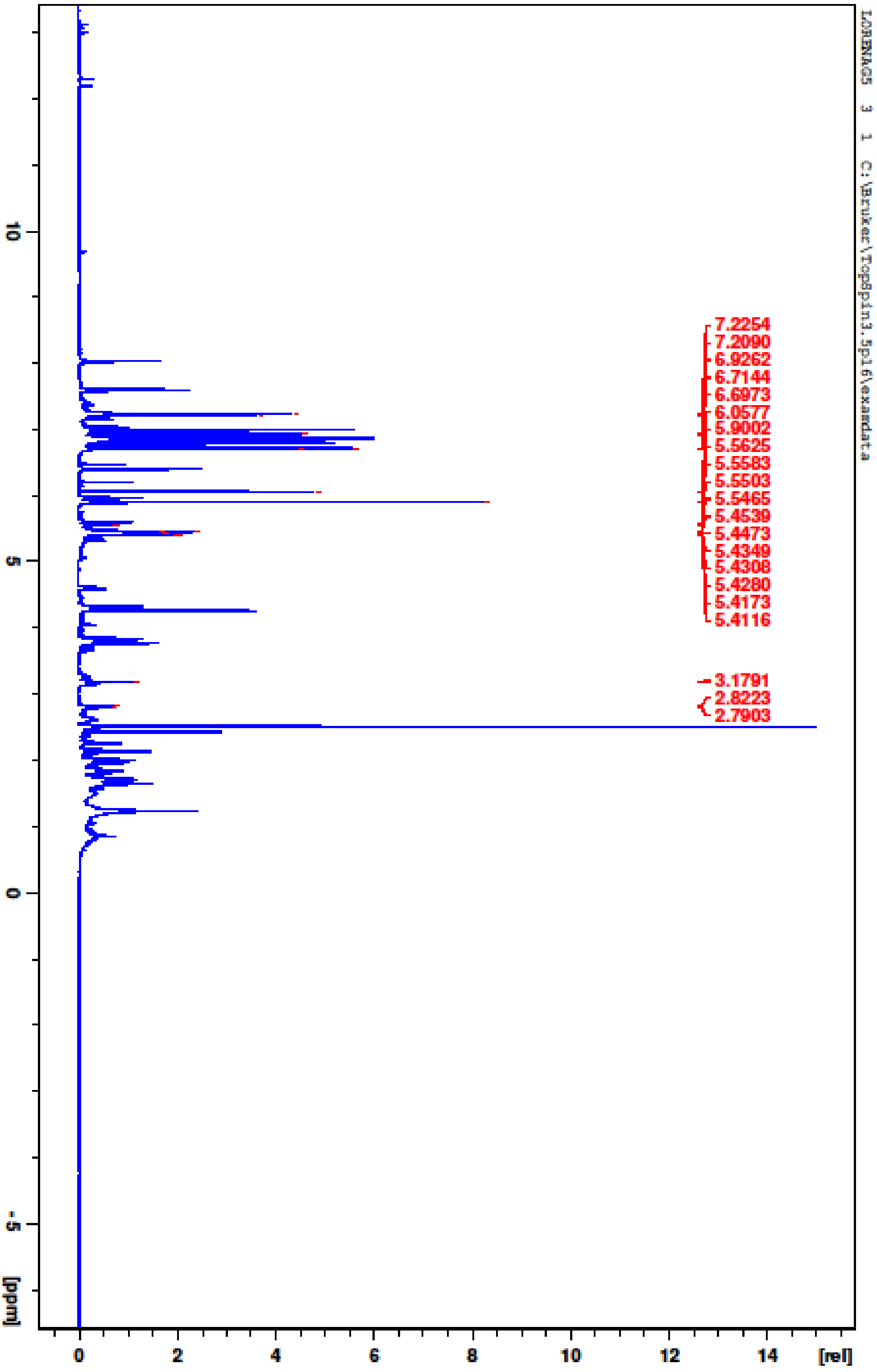

Figure S1: ^{1}H NMR spectrum of compound **1** and **2** (DMSO-d_6, 500 MHz).

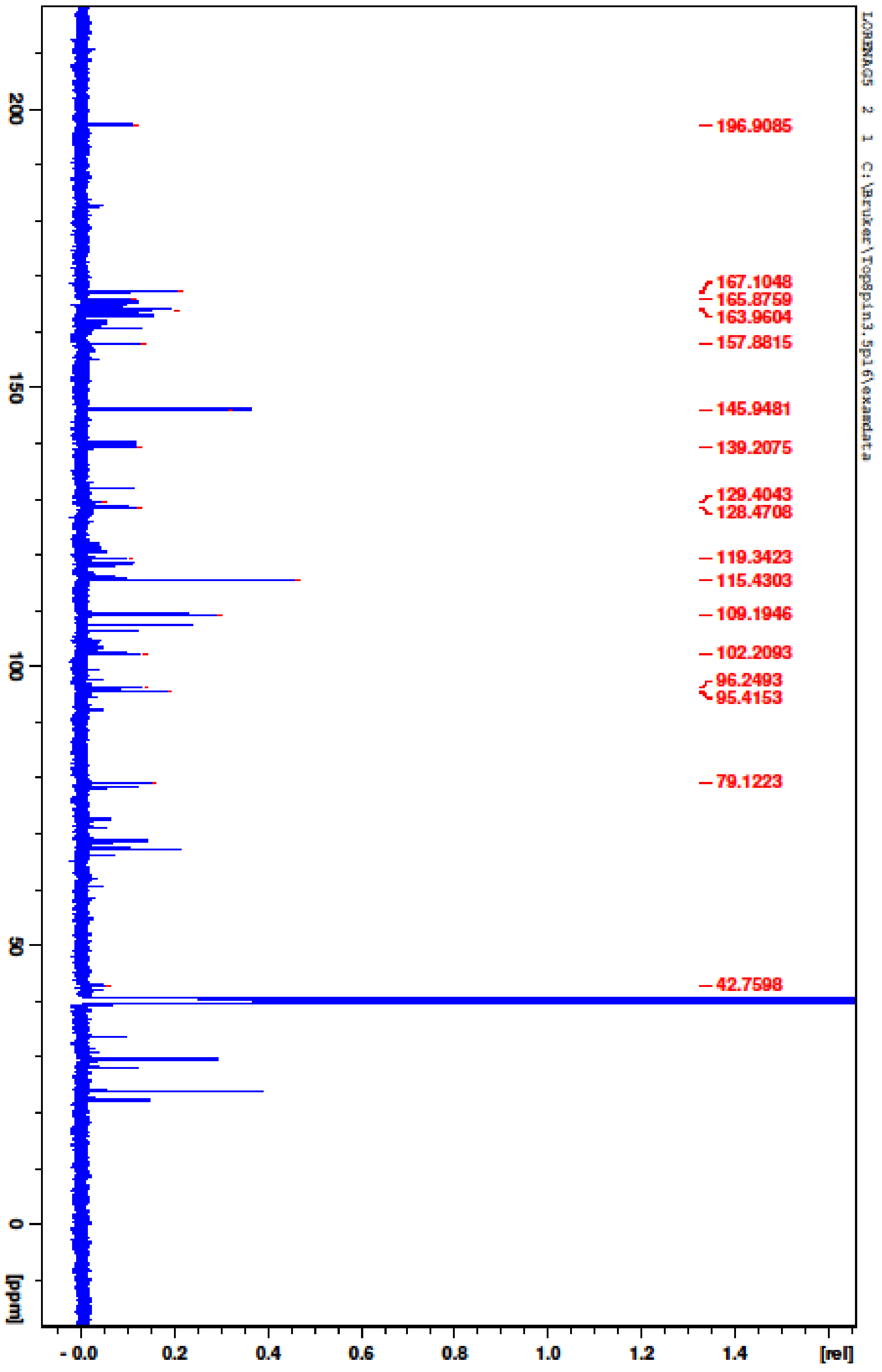

Figure S2: ^{13}C NMR spectrum of compound **1** and **2** (DMSO-d_6, 125 MHz).

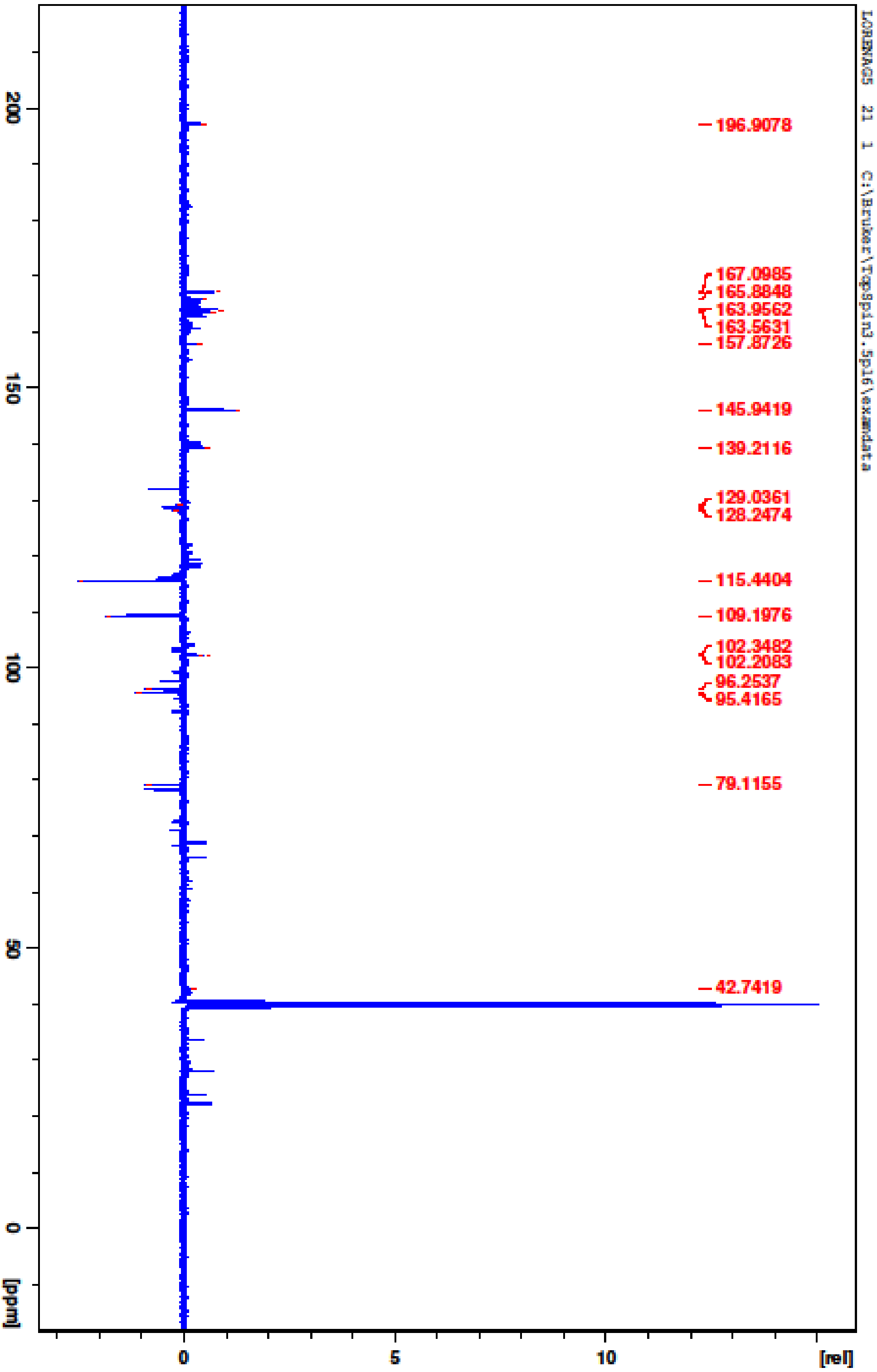

Figure S3: ^{13}C DEPTQ NMR spectrum of compound **1** and **2** (DMSO-d_6, 125 MHz).

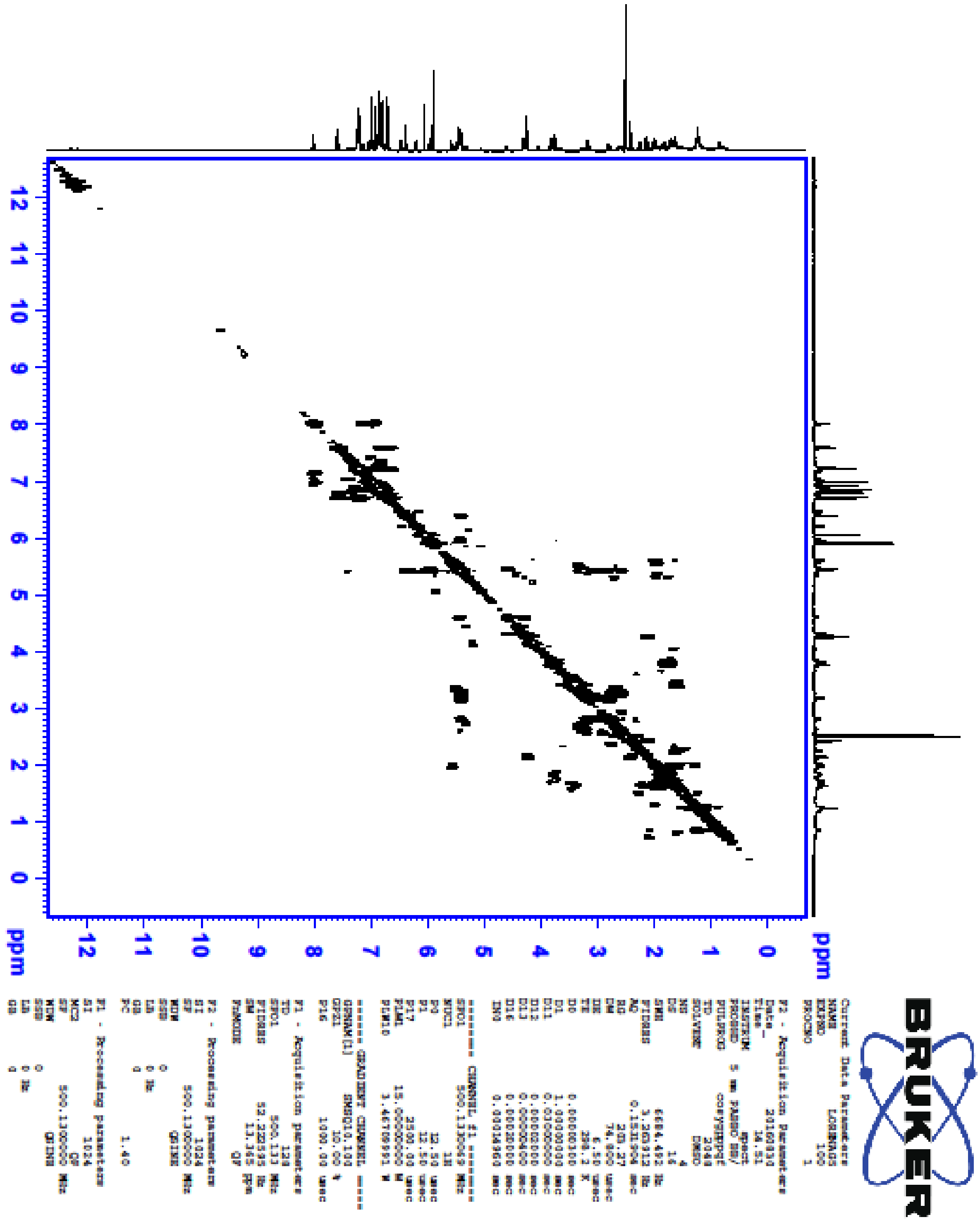

Figure S4: COSY spectrum of compound **1** and **2** (DMSO-d_6, 500 MHz).

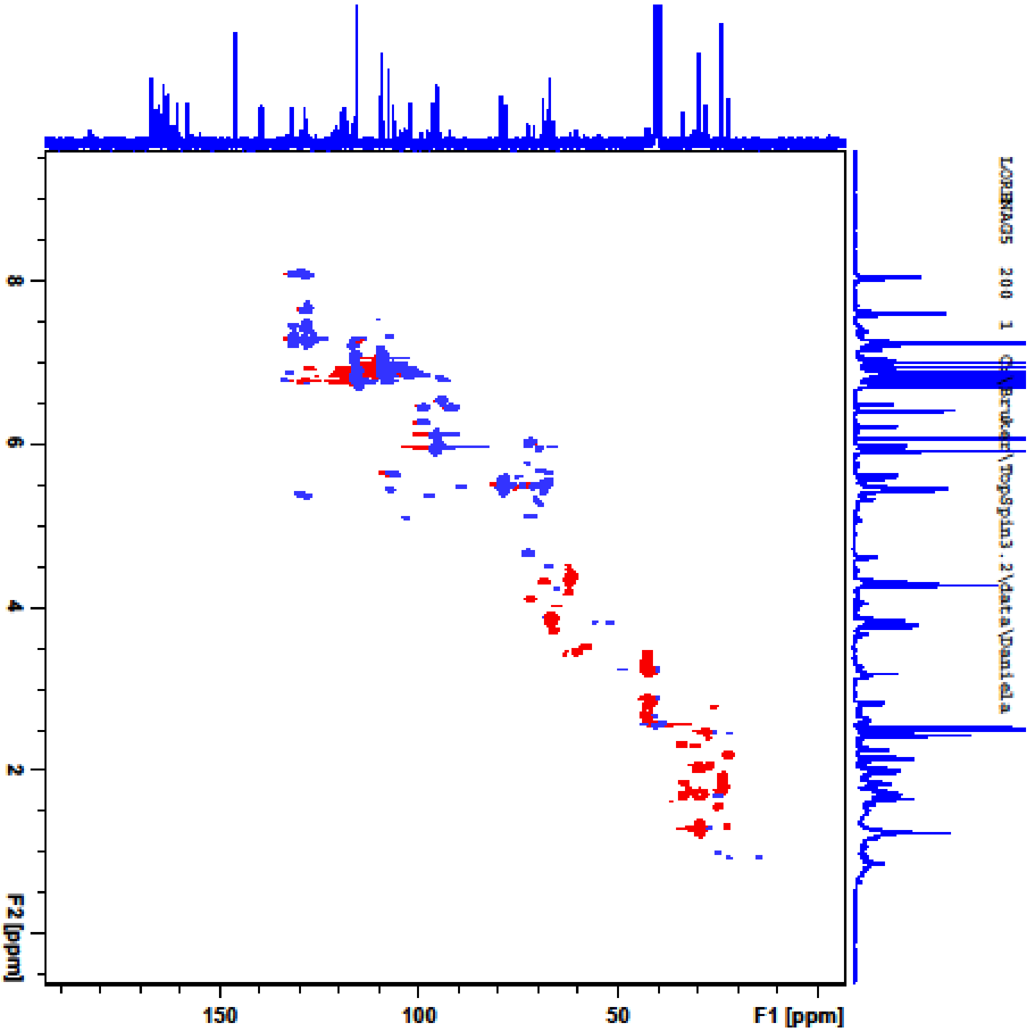

Figure S5: HSQC spectrum of compound **1** and **2** (DMSO-d_6, 500 MHz).

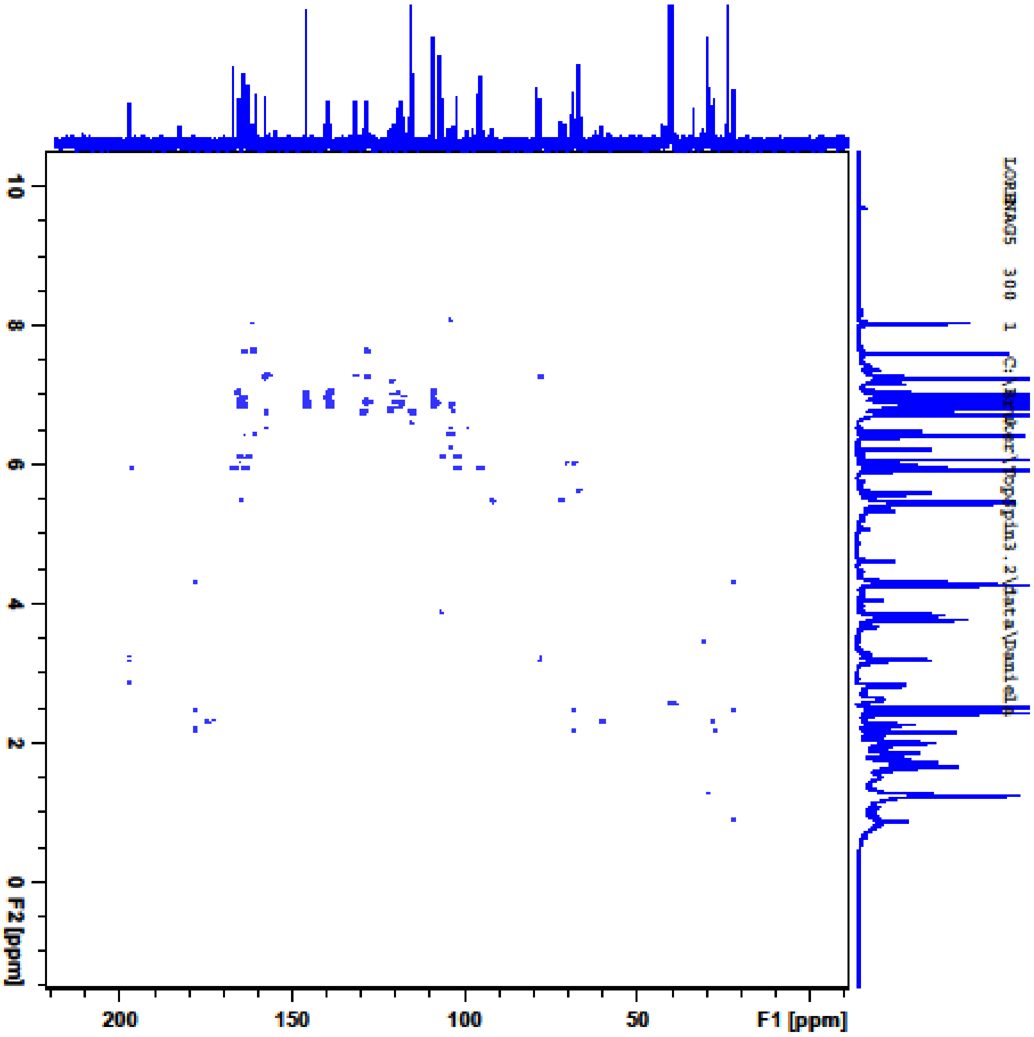

Figure S6: HMBC spectrum of compound **1** and **2** (DMSO-d_6, 500 MHz).

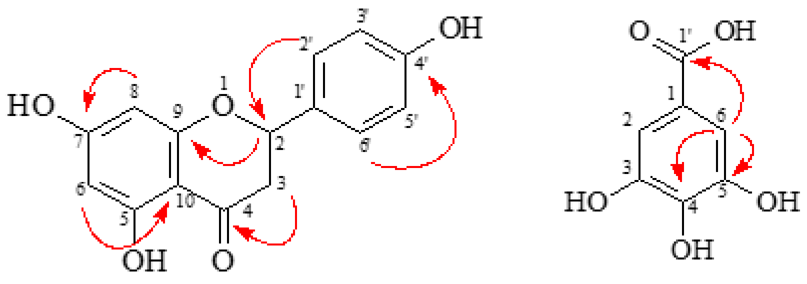

1 **2**

Figure S7: Structures of compound **1** and **2** assembled with the aid of COSY correlations.
